# Effect of Two-Step Austempering Process on Transformation Kinetics of Nanostructured Bainitic Steel

**DOI:** 10.3390/ma12010166

**Published:** 2019-01-07

**Authors:** Chunhe Chu, Yuman Qin, Xuemei Li, Zhinan Yang, Fucheng Zhang, Changhong Guo, Xiaoyan Long, Leilei You

**Affiliations:** 1 State Key Laboratory of Metastable Materials Science and Technology, Yanshan University, Qinhuangdao 066004, China; chuchunheysu@sina.com (C.C.); qinyuman0325@foxmail.com (Y.Q.); longxiaoyanlxy@163.com (X.L.); 2 National Engineering Research Center for Equipment and Technology of Cold Strip Rolling, Yanshan University, Qinhuangdao 066004, China; junan961@163.com; 3 School of Mechanical Engineering, Yanshan University, Qinhuangdao 066004, China; guochanghong@ysu.edu.cn; 4 Luoyang LYC Bearing Co., Ltd., Luoyang 471039, China; youlei105@sina.com

**Keywords:** nanostructured bainite, bearing steel, two-step austempering process, phase transformation

## Abstract

The two-step austempering process has been reported to be an effective method to accelerate the bainitic transformation process by introducing martensite (Q-M-B). However, in this study, it was found that the Q-M-B process reduced the incubation time, but the transformation duration remained nearly unchanged. The notably reduced activation energy barrier for nucleation of bainitic ferrite on the preformed martensite should be responsible for the reduced duration time of the Q-M-B process. A process that both of the two steps were above, Ms (Q-B-B), has been demonstrated to increase transformation rate and improve the amount of bainitic ferrite, which probably results from the additional hysteresis free energy provided by the first quenching process.

## 1. Introduction

The bearing is a basic and key component in many mechanical parts, and bearings with the required properties are necessary for steady operation of the machinery. Therefore, improving the properties of bearing steel has been the goal of material researchers. Nanostructured bainitic microstructure not only possesses excellent strength and toughness [1,2,3,4,5], but exhibits a high wear resistance and has a better rolling contact fatigue performance compared to the martensitic microstructure [6,7,8,9,10,11]. This material is therefore suitable for the bearing application [12,13,14].

To obtain the nanostructured bainitic microstructure and a hardness of at least 58 HRC, the steel should be austempered at a lower temperature [15]. This inevitably results in a long austempering time, which is not acceptable to the bearing industry. To accelerate kinetics of the bainitic phase transformation, researchers have developed methods such as adding Co and Al [16,17], or reducing Mn [18] in the steel to enlarge the free energy difference, thus obtaining a higher driving force for phase transformation. Other approaches include introducing extra stress that is lower than the yield stress, or introducing a magnetic field to induce phase transformation [19,20]. Carrying out the ausforming process before the bainitic phase transformation can also notably influence the kinetics, which are determined by the ausforming temperature and extent of deformation [21,22]. However, at present, these methods are not suitable for the heat treatment of bearing materials.

Studies have demonstrated that a two-step treatment within the bainite transformation range can shorten the duration of bainitic phase transformation of high carbon bearing steel and also guarantee its hardness [23,24,25]. Vetters et al. also revealed that the transformation of 100Cr6 bearing steel could be accelerated by introducing a certain amount of martensite before the bainitic phase transformation [25]. However, this process has not been widely investigated in bearing steel. Some studies on other steels have shown that introducing a certain amount of martensite could accelerate the transformation process [26,27,28]. The microstructure is also influenced by the pre-formed martensite [26]. Interestingly, Smanio and Sourmail found that the kinetics of bainite formation after prior formation of a given fraction of martensite were identical to those observed after formation of the same fraction of bainite [27]. This was also demonstrated by Gong et al. [26].

Recently, the authors found that introducing a certain amount of martensite before the bainitic phase transformation at low temperature notably reduced the incubation time, but this process showed a negligible effect on the duration time. Moreover, it was interesting to discover that maintaining a temperature slightly above the martensite start (Ms) temperature, but lower than the austempering temperature, could also shorten the incubation time and promote phase transformation. 

In this paper, some two-step treatments were carried out on a nanostructured bainitic bearing steel. The transformation kinetics were analyzed with the aim of revealing the effect of these treatments on the phase transformation of nanostructured bainitic bearing steel. Moreover, its applicability was also addressed via the hardness test.

## 2. Experimental Procedures

**Figure 1:**
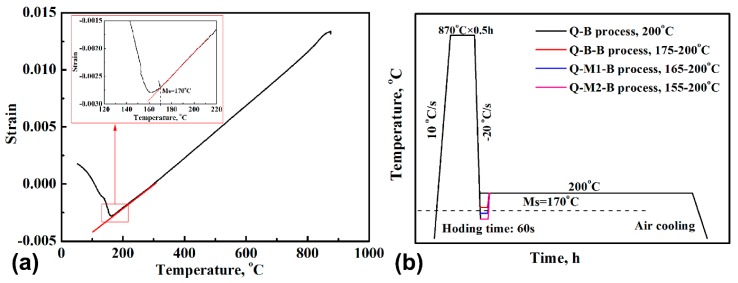
The strain-temperature curve for determining the (**a**) Ms temperature and (**b**) detailed heat treatment process.

The chemical composition of the nanostructured bainitic bearing steel used for this study was Fe-0.83C-0.65Mn-1.40Si-1.62Cr-0.31Ni-0.17Mo (wt. %). The steel was melted in a vacuum induction furnace and hot-forged into a round bar. The steel was then tempered at 650 °C for 3 h. The Ms temperature of the tempered steel was measured using a DIL 805A dilatometer (TA, Hüllhorst, Germany), and it was determined to be 170 °C, as shown in Figure 1a. According to the Ms temperature, three types of heat treatment processes were designed. After austenitizing at 870 °C for 0.5 h, the specimens were treated by different processes as follows: (1) directly austempering at 200 °C (Q-B), (2) first quenching to a temperature of 175 °C for 60 s and then increasing the temperature to 200 °C (Q-B-B), or (3) first quenching to a temperature of 165 °C or 155 °C for 60 s and then increasing the temperature to 200 °C (Q-M-B). The increasing rate from the first step temperature to 200 °C is 10 °C/s and the holding time for austempering at 200 °C is 30 h. The detailed process is described in Figure 1b. The kinetic curves for phase transformation of the steel were also recorded with a DIL 805A dilatometer.

The amount of retained austenite for each specimen was analyzed by a D/max-2500/PC X-ray diffractometer (XRD, Rigaku, Tokyo, Japan) with a scanning rate of 2° min^−1^ over 2θ of 40–100° and unfiltered CuKα at 40 kV and 200 mA. The specimens were sectioned transversely and polished following a conventional metallographic technique. A 4% Nital etchant was used to reveal the microstructure. Microstructural characterizations were carried out by SU-5000 type thermal-emission scanning electron microscope (SEM, Hitachi, Tokyo, Japan). The hardness of the specimens after tempering was determined using a HR-150A Rockwell hardness tester (Huayin test, Yantai, China), and the measurements were repeated five times to obtain an average value.

## 3. Results

**Figure 2:**
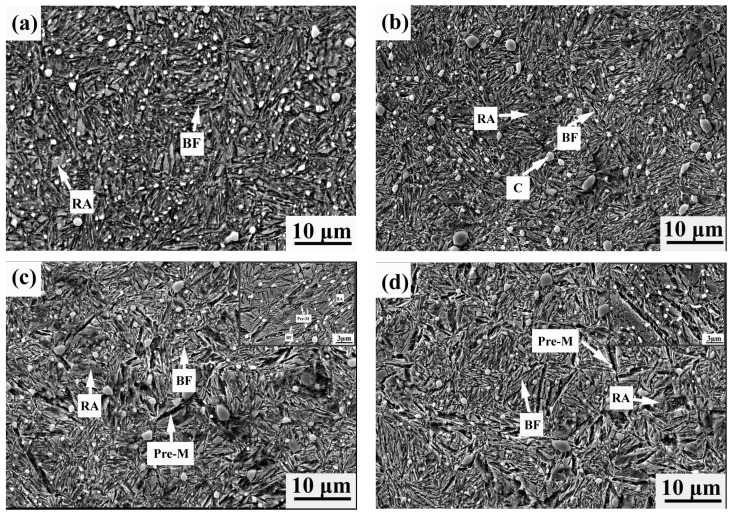
SEM observations of the specimen with different treatment process, (**a**) Q-B process, (**b**) Q-B-B, (**c**) Q-M1-B process, (**d**) Q-M2-B process. Note: BF, RA, Pre-M, and C represent bainitic ferrite, retained austenite, preformed martensite and cementite, respectively.

Figure 2 shows the microstructure of specimens after different treatment processes. Obviously, there is an abundance of cementite dispersed within the microstructure of all specimens, and statistic results reveal that the fraction of cementite is ~6.5% in all specimens for the same high temperature temper process and the subsequent austenitization process. There is preformed martensite in the microstructures of the Q-M1-B and Q-M2-B specimens, and the amount of martensite is higher in the Q-M2-B specimen than that of the Q-M1-B specimen due to a lower quenching temperature. Moreover, it can be seen that there are bainitic ferrite plates originated from the preformed martensite, indicating that it provided a nucleation site for the bainitic ferrite. It also seems that the bainitic ferrite in the Q-B-B specimen is finer and shorter than that of the Q-B specimen, as shown in Figure 2a,b. This suggests that the nucleation site for bainitic ferrite in the Q-B-B specimen is higher than that of the Q-B specimen, which not only refines the microstructure, but increases the impingement chance of bainitic ferrite sheaves and then shortens the bainitic ferrite plate. This will also be discussed in Section 4.2.

**Figure 3:**
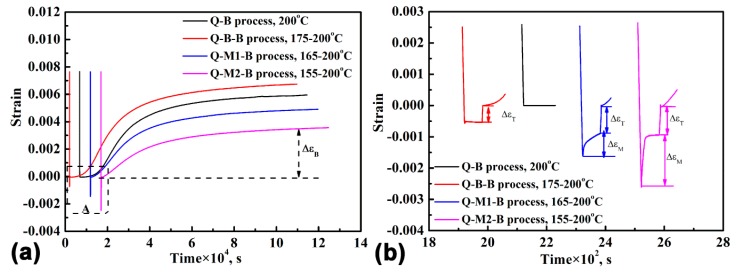
(**a**) Kinetic curves for phase transformation for the specimens with different processes, (**b**) the enlarged view of region A. Note: $\Delta \varepsilon_{T},\;\Delta \varepsilon_{B}\;\mathrm{and}\;\Delta \varepsilon_{M}\;$ represent the strain results from temperature, bainite and martensite, respectively.

Figure 3 shows the kinetic curve of each specimen during the heat treatment process and an enlarged view of the initial stage. When the holding time at 200 °C reached 30 h, the increase of the strain rate became very slow and was nearly the same for all specimens, as shown in Figure 3a. The slow increase in strain rate indicated that the transformation from austenite to bainitic ferrite became sluggish. Therefore, the tests were stopped when the holding time reached 30 h. The dilatation strain during the final cooling process was also recorded, which verified that no martensite transformation occurred during the final cooling after the austempering process. Moreover, the kinetic curve indicated that the strain ($\Delta \varepsilon_{B}$) resulting from the formation of bainitic ferrite was much smaller for the Q-M2-B specimen than for the other three specimens. 

From the enlarged view of the initial stage of the kinetic curves that are shown in Figure 3b, it can be seen that there are dilatational strains ($\Delta \varepsilon_{M}$) for both Q-M1-B and Q-M2-B specimens during the cooling process from the austenitizing temperature to the first holding temperature. The dilatational strains were caused by the martensitic transformation. The $\Delta \varepsilon_{M}\;$ is larger for the lower quenching temperature of the first process. The volume fraction of pre-formed martensite (*V_M_*) during the cooling process can be estimated according to the Koistinen–Marburger relationship, where *T_q_* is the holding temperature during the cooling process. [29]:

(1) $$V_{M}=1-e^{-1.1\times 10^{-2}\left(M_{s}-T_{q}\right)}$$

The calculated results are shown in Table 1, which shows that there was 5.4 vol. % and 15.2 vol. % martensite formed during the cooling process for the Q-M1-B specimen and Q-M2-B specimen, respectively. For the Q-B-B specimen, there was no further increase of the strain during the short holding at the first temperature, revealing that no phase transformation occurred during this process. During the increasing temperature process, there were dilatational strains ($\Delta \varepsilon_{T}$) that likely resulted from the thermal expansion.

The incubation time for the formation of bainitic ferrite can also be determined from the kinetic curves, as shown in Table 1. It can be seen that the incubation time gradually decreased from 1324 s to 40 s as the first holding temperature decreased. Many studies demonstrated that pre-formed martensite can notably shorten the incubation time of the bainite transformation [26,27,28,30]. However, this is the first evidence that the incubation time can also be effectively shortened by the Q-B-B process and without introducing pre-formed martensite and bainitic ferrite. The reduction reaches 35% for the Q-B-B process.

**Figure 4:**
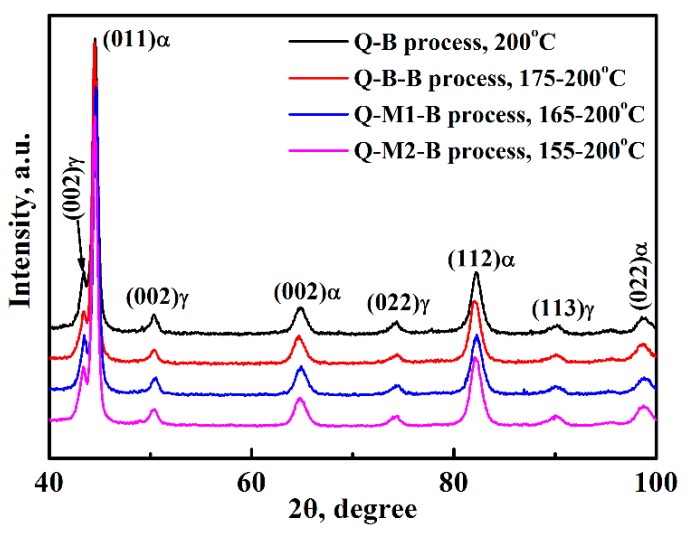
XRD patterns for the specimens treated by different processes.

Figure 4 shows the XRD patterns for these specimens after different treatment processes. Two types of diffraction peaks can be observed, namely the α phase with the body centered cubic structure and the γ phase with the face centered cubic structure. The fraction of cementite was ~6.5 vol. %. According to the diffractions’ patterns, the volume fraction of retained austenite can be estimated, and the results are shown in Table 1. One can see that the volume fraction of retained austenite in Q-B, Q-M1-B and Q-M2-B specimens is similar, and it is lowest for the Q-B-B specimen. The volume fraction of bainitic ferrite can also be calculated, as shown in Table 1. The volume fraction of the bainitic ferrite plus martensite is more voluminous for the Q-B-B specimen. This result also indicates the transformation of undercooled austenite is more sufficient for the Q-B-B process than for the conventional Q-B process and the new Q-M-B process. Combining the SEM observations, it can be concluded that the Q-B-B process not only shortens the incubation time but also improves the transformation process when considering the previous results for incubation time.

**Figure 5:**
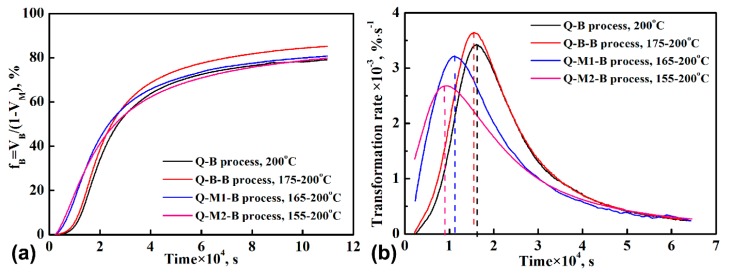
(**a**) Variation of volume fraction of bainitic ferrite with holding time and (**b**) the variation of transformation rate with holding time.

Figure 5 shows the variation of the normalized volume fraction of bainitic ferrite, (*f*_B_ = *V*_B_/(1 − *V*_M_)), and the corresponding transformation rate as a function of increasing holding time. During the initial holding process at 200 °C, the transformation rate is notably higher for the two Q-M-B processes. It is the highest for the Q-M2-B process as compared to the Q-B and Q-B-B processes. However, for the two Q-M-B processes, the transformation rate reached the peak value very quickly. Moreover, for the Q-M2-B process, the peak value of the transformation rate is smaller and the time needed for reaching the peak value is shorter. For the two processes without pre-formed martensite, the transformation rate was always slightly higher for the Q-B-B process than for the Q-B process. The peak values for the transformation rate of Q-B and Q-B-B processes are higher than the two Q-M-B processes, as shown in Figure 5b. It can also be seen that the transformation rate of the Q-B and Q-B-B processes are higher than the two Q-M-B processes after the peak rate. Moreover, the final transformation rate for the four processes is nearly the same. The higher transformation rate for the Q-B-B process is likely responsible for the improved transformation extent of the austenite.

The hardness of the specimens was measured and the results are shown in Table 2. It can be observed that the hardness for all specimens is slightly higher than 60 HRC, which meets the hardness requirement for bearings. This demonstrates that all four of these processes are candidates for the heat treatment of bearings.

## 4. Discussion

### 4.1. Effect of Q-M-B Process on the Bainitic Phase Transformation

Several studies have demonstrated that introducing a certain amount of martensite can accelerate the following bainitic phase transformation process [26,27,28,30]. Gong et al. considered that dislocations introduced in austenite due to the stress relaxation of the transformation strains could assist the bainite transformation [26]. Kawata et al. suggested that the boundary between martensite and austenite was a prior nucleation site of bainitic ferrite and should be responsible for the accelerated transformation process [30]. The transformation process of bainite includes the nucleation of bainitic ferrite and the sequenced growth of it. The present results revealed that the Q-M-B process notably shortens the incubation time of the bainitic transformation, which is consistent with the above-mentioned results. However, the mechanism has not yet been clarified. 

The shortened incubation time for the Q-M-B process revealed that the nucleation process became easier due to the existence of martensite. This indicates that the activation energy barrier for nucleation, $G^{*}$, is lower when the nucleation occurs at the interface of austenite and martensite. According to classical nucleation theory, the activation energy barrier for nucleation, $G^{*}$, can be given by [31]:

(2) $$G^{*}=\frac{16\pi \sigma_{\alpha /\gamma }^{3}}{3{\left(\Delta G_{chem}+\Delta G_{strain}\right)}^{2}}$$

where $\Delta G_{chem}=G_{V}^{\alpha }-G_{V}^{\gamma }$, $G_{V}^{\alpha }$ and $G_{V}^{\gamma }$ is the Gibbs free energy per unit volume of ferrite (α) and austenite (γ), respectively, $\Delta G_{strain}$ is the strain energy per unit volume of α and $\sigma_{\alpha /\gamma }$ is the interfacial energy between α and austenite (γ). If the nucleation is located in the austenite/martensite interface, the interfacial energy should be $\sigma_{\alpha /M}$. The $\Delta G_{chem}$ and $\Delta G_{strain}$ is the same for both nucleation at the interface of austenite/martensite and at grain boundary of austenite. Therefore, the interfacial energy was the main difference between the two nucleation modes.

The interfacial energy is determined by the crystal structure of the neighboring phases and decreasing the mismatch of the neighboring phases can reduce it. The interface between martensite and ferrite is coherent, and the interfacial energy was determined to be 0.016 J/m^2^ [32]. The interface between austenite and ferrite is semi-coherent, and the interfacial energy between them was determined to be 0.20 J/m^2^ [33]. Taking the interfacial energy in to Equation (3), the following relationship can be obtained:

(3) $$G_{\alpha /M}^{*}=0.000512G_{\alpha /\gamma }^{*}$$

Therefore, the activation energy barrier for the nucleation of bainite ferrite at the phase boundary of austenite/martensite was only 0.000512 times as much as at an austenite grain boundary. Moreover, the martensite transformation during the first step inevitably introduces dislocations at the interface of the austenite and martensite. The nucleation at dislocations can relax the elastic strain energy of partial dislocations [26] and then further reduce the activation energy barrier. It is concluded that the lowered activation energy barrier promotes the nucleation process of the bainitic ferrite and then shortens the incubation time.

More bainitic ferrite continued to be formed as the holding time increased, which increased the transformation rate due to the promoted autocatalysis effect. However, the bainite cannot cross the interface and grain boundaries [31]. The pre-formed martensite indirectly refined the grain size and then impeded the growth of the bainite. Therefore, the maximum transformation rate is lower for the Q-M-B process than for the Q-B and Q-B-B processes, as shown in Figure 5b. The maximum transformation rate decreases with an increasing amount of pre-formed martensite. Moreover, the dislocations introduced by martensite improve the stability of the untransformed austenite, which inevitably reduces the transformation rate. These factors together result in the transformation time for the specimen treated by the Q-M-B process to not be shortened. 

### 4.2. Effect of Q-B-B Process on the Bainitic Phase Transformation

**Figure 6:**
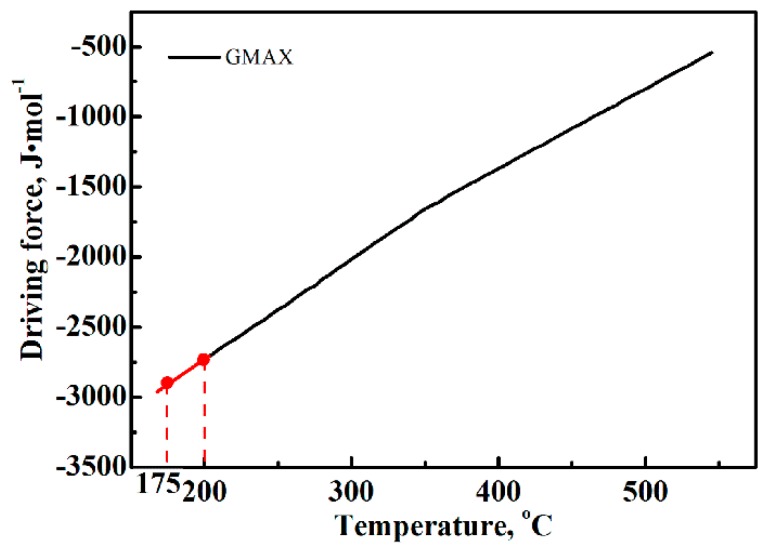
Estimated free energy difference according to the MUGG83 model. Note: GMAX is the maximum of free energy difference.

The free energy difference (∆G) between austenite and ferrite provides the driving force for the transformation. Upon decreasing the temperature, the ∆G increases and it increases the driving force for the transformation. According to the MUGG83 thermodynamic model (MUGG83, Mathew Peet and H.K.D.H. Bhadeshia, Cambridge, UK) [34,35], the ∆G for the nucleation of bainitic ferrite of the present steel can be estimated, as shown in Figure 6. It can be observed that with decreasing temperature, the ∆G increases in a nearly linear fashion. Therefore, the ∆G value at 175 °C for the present steel can be estimated by extension of the line, even though the lowest temperature in this mode is 200 °C. Using this approach, the ∆G_200_ and ∆G_175_ were determined to be −2720 J/mol and −2920 J/mol, respectively.

**Figure 7:**
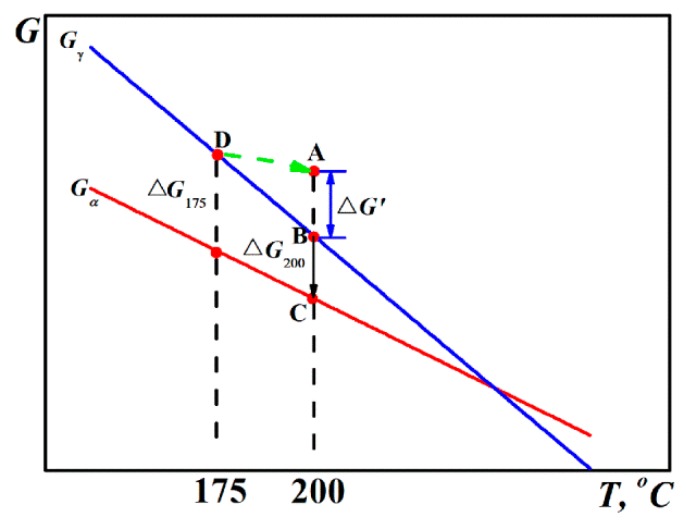
Evolution of free energy of austenite (G_γ_) and ferrite (G_α_) with decreasing temperature.

For the Q-B-B process, the specimen was held at 175 °C for 60 s, and then the temperature was increased to 200 °C at a rate of 10 °C/s. The SEM observation and transformation kinetics result have demonstrated that the followed transformation process was accelerated and improved as compared to the conventional Q-B process, indicating that the driving force at 200 °C is higher than −2720 J/mol and there exists an additional driving force due to the first step treatment. It is probably the hysteresis free energy, ∆G’, due to the thermal hysteresis effect taking place when the specimen was heated to 200 °C from 175 °C, as shown in Figure 7. Moreover, the hysteresis free energy should be influenced by the heating rate, where a faster heating rate results in a higher hysteresis free energy. The increased free energy change provided a larger driving force for transformation, which inevitably increased the amount of nucleation site for bainitic ferrite and then accelerated the transformation process. This resulted in a reduced amount of retained austenite and an increased amount of bainitic ferrite formed at the same austempering time compared to the Q-B specimen, as shown in Table 1.

## 5. Conclusions

The effect of two-step treatments on the bainitic transformation of nanostructured bainitic bearing steel was studied and the following results were obtained:

1. Introducing pre-formed martensite by the Q-M-B process notably reduced the incubation time for bainitic transformation. The lowered activation energy barrier for bainite at the interface of martensite/austenite was only 0.000512 times as much as at the austenite grain boundary. However, the whole transformation time was not shortened due to the reduced transformation rate at a later stage. The reduction in incubation time and transformation rate at a later stage increased with an increasing amount of pre-formed martensite.
2. As compared to the conventional Q-B process, the Q-B-B process also shortened the incubation time and improved the transformation of austenite to bainite. The additional hysteresis free energy introduced by fast heating from the lower temperature (175 °C to 200 °C) should be responsible for the positive impact of the process.
3. The hardness of all of these specimens treated by the different processes was higher than 60 HRC and met the hardness requirement for bearings.

## Figures and Tables

**Table 1:** The incubation time and microstructure parameters for specimens treated by different processes.

Process	Incubation Time, s	Volume Fraction of Each Phase, vol. %			
		Martensite	Retained Austenite	Bainitic Ferrite	Cementite
Q-B	1324	0	14.8	78.7	6.5
Q-B-B	864	0	8.7	84.8	6.5
Q-M1-B	101	5.4	12.2	75.9	6.5
Q-M2-B	40	15.2	11.1	67.2	6.5

**Table 2:** Hardness result for the specimens treated by different processes.

Process	Q-B	Q-B-B	Q-M1-B	Q-M2-B
Hardness	60.7 ± 0.2 HRC	60.2 ± 0.2 HRC	60.4 ± 0.3 HRC	60.3 ± 0.4 HRC
